# Mining data and metadata from the gene expression omnibus

**DOI:** 10.1007/s12551-018-0490-8

**Published:** 2018-12-29

**Authors:** Zichen Wang, Alexander Lachmann, Avi Ma’ayan

**Affiliations:** BD2K-LINCS Data Coordination and Integration Center; Knowledge Management Center for the Illuminating the Druggable Genome; Mount Sinai Center for Bioinformatics, Department of Pharmacological Sciences, Icahn School of Medicine at Mount Sinai, Box 1603, One Gustave L. Levy Place, New York, NY 10029 USA

**Keywords:** GEO, Gene Expression Omnibus, Computational data curation, Natural language processing, FAIR principles

## Abstract

Publicly available gene expression datasets deposited in the Gene Expression Omnibus (GEO) are growing at an accelerating rate. Such datasets hold great value for knowledge discovery, particularly when integrated. Although numerous software platforms and tools have been developed to enable reanalysis and integration of individual, or groups, of GEO datasets, large-scale reuse of those datasets is impeded by minimal requirements for standardized metadata both at the study and sample levels as well as uniform processing of the data across studies. Here, we review methodologies developed to facilitate the systematic curation and processing of publicly available gene expression datasets from GEO. We identify trends for advanced metadata curation and summarize approaches for reprocessing the data within the entire GEO repository.

## Introduction

**Fig. 1:**
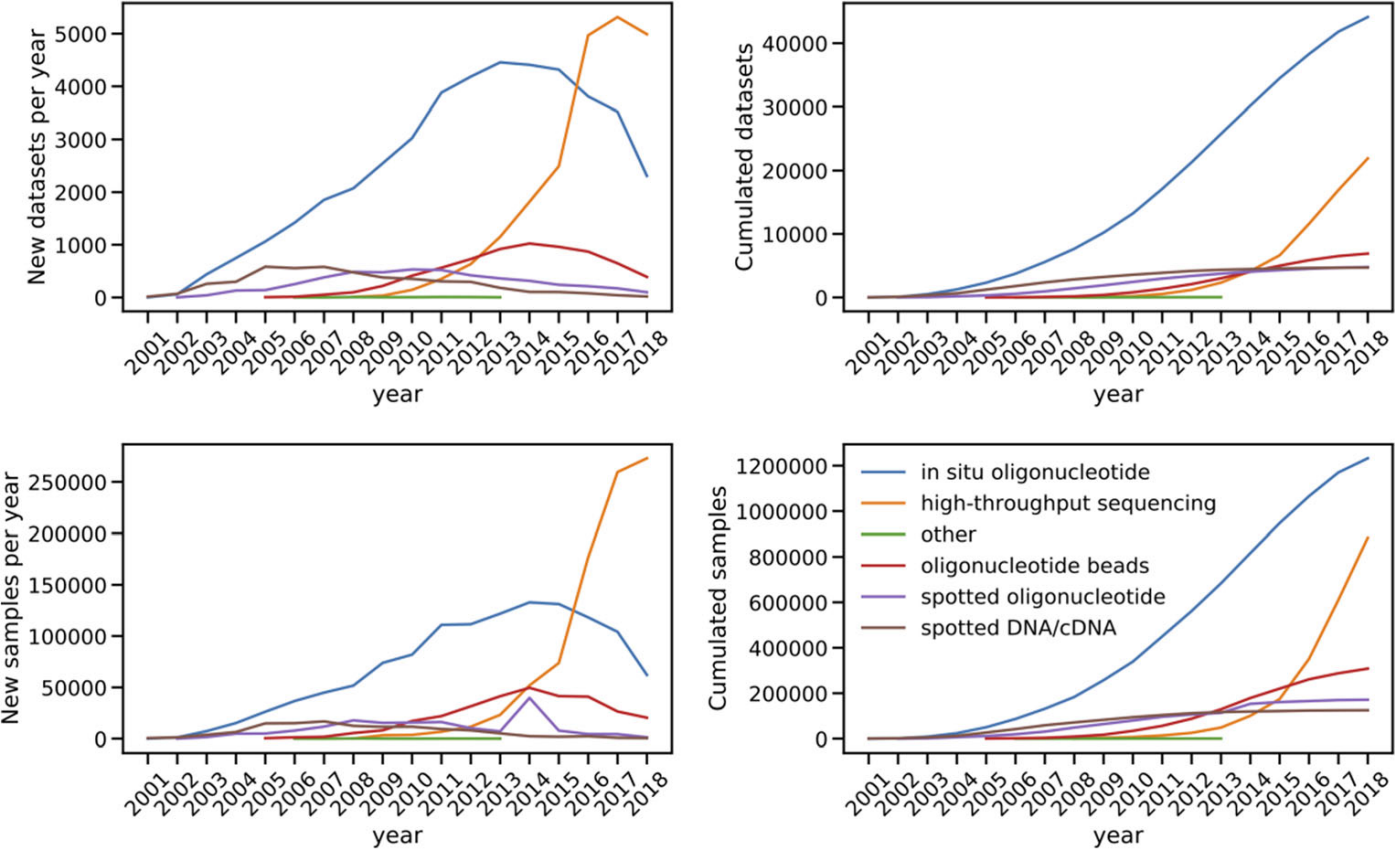
The growth of publicly available gene expression datasets and samples from GEO over time. Plots on the top panel show the growth of gene expression datasets from different transcriptomic profiling technologies over time, whereas plots on the bottom panel show the growth of individual samples from those datasets. The plots were made on September 2018. Hence, the total for 2018 cover only part of the year

Gene expression datasets are accumulating rapidly in public repositories such as the NCBI’s Gene Expression Omnibus (GEO) (Barrett et al. 2013) and the Sequence Read Archive (SRA) (Kodama et al. 2012) as well as ArrayExpress (Rustici et al. 2013). That is partly driven by the emergence of new and improved transcriptomic profiling technologies such as RNA sequencing (RNA-seq) (Fig. 1). In addition, most journals now mandate the deposition of transcriptomics data as a requirement for publication, with the goal of enabling reproducibility and data reuse. Reanalysis and integration of themed collections of gene expression datasets can produce new insights into the underlying biological mechanisms under investigation. For instance, meta-analysis of multiple datasets for a disease can help in discovering the most consistently differentially expressed genes (DEGs) and the pathways that these genes belong. In addition, consistent DEGs can become biomarkers and drug targets. Similarly, curated collections of gene expression signatures can serve as a Connectivity Map reference database for matching user-submitted signatures of DEGs with annotated and curated signatures (Lamb et al. 2006; Subramanian et al. 2017). Similarly, curated signatures can be converted to gene set libraries for gene set enrichment analyses (Chen et al. 2013; Kuleshov et al. 2016; Subramanian et al. 2005). In addition, curated signatures can be compared for reproducibility across multiple independent studies (Gundersen et al. 2016), or for finding unexpected relationships between drugs, genes, and diseases (Wang et al. 2016; Chen and Butte 2016; Cheng et al. 2014).

Several software tools have been developed for reanalyzing individual or collections of datasets from GEO (Table 1). Those tools enable users to search GEO for relevant studies and then retrieve specific datasets for further analysis. In addition to those tools, approaches have been developed to uniformly reprocess all the microarray or RNA-seq datasets in GEO. The uniformly reprocessed gene expression datasets can be organized into databases that serve as search engines that enable knowledge discovery at the data level. Prominent examples include ExpressionBlast (Zinman et al. 2013), Recount2 (Collado-Torres et al. 2017), ARCHS4 (Lachmann et al. 2018), and SEEK (Zhu et al. 2015). These resources processed a large number of microarray and RNA-seq samples to build search engines for gene expression profiles and co-expression modules. Recent advances in cloud computing infrastructure, efficient cloud-enabled aligners such as Rail-RNA (Nellore et al. 2017), and alignment-free RNA-seq quantification methods such as Kallisto (Bray et al. 2016) enable the large-scale uniform reprocessing of RNA-seq datasets from GEO. Such efforts include Recount2 (Collado-Torres et al. 2017) and ARCHS4 (Lachmann et al. 2018). These newer search engines provide other features besides sample search, for example, gene function prediction, average expression in tissues and cells, and systematic discovery of alternative splicing events.

**Table 1:** Software tools developed for reanalyzing and further annotating GEO datasets

Tool	Citation	Individual/multiple	Type	Note	Limitations
GEO2R	(Barrett et al. 2013)	Individual	Web	Implements GUI that generate graphs and R script	Limited graphical visualizations; only implements DE analysis; limited to microarray data
shinyGEO	(Dumas et al. 2016)	Individual	Web	R Shiny extension of GEO2R with improved graphics	DE analysis only available for individual genes; limited to microarray data
GEOquery	(Davis and Meltzer 2007)	Individual	R package	Bridge between GEO and BioConductor to enable analyses of GEO datasets in various BioConductor packages	Requires users to be proficient in R and Bioconductor packages; limited to microarray data
GEO2Enrichr	(Gundersen et al. 2015)	Individual	Brower extension	Identifies DEGs and pipe to enrichment analysis tool	Limited to microarray data; limited analysis components
BioJupies	(Torre et al. 2018)	Individual	Web	Generates interactive Jupyter notebooks from RNA-seq datasets	Limited to RNA-seq data. Only allows 2 group comparison
ScanGEO	(Koeppen et al. 2017)	Multiple	Web	Identifies DEGs across multiple GEO studies matching user-specified criteria	Limited to curated GEO datasets (GDS); only supports DE analysis
ImaGEO	(Toro-Domínguez et al. 2018)	Multiple	Web	Performs nine types of meta-analysis across multiple GEO studies	Limited to microarray datasets
GEOracle	(Djordjevic et al. 2017)	Multiple	Web	Uses text mining of the GEO metadata to automatically identify perturbational GEO datasets and associated metadata	Limited to microarray datasets; only performs DE analysis

However, integrating datasets across studies as well as performing meta-analyses from collections of studies is still difficult. This is mainly because of the lack of machine-readable standardized metadata at the study and sample levels. The metadata associated with gene expression studies within GEO typically do not adhere to controlled vocabularies to describe biological entities such as tissue type, cell type, cell line, gene/protein, drug/small-molecule, and disease. Instead, the authors of the datasets use semi-structured textual descriptions to annotate their study design, sample characteristics, and experimental protocols. Many GEO studies are also associated with publications indexed in PubMed, which further helps other researchers to understand the details of each study design, but does not resolve the necessity for machine-readable metadata.

Therefore, there is an urgent need for better curating and annotating publicly available gene expression datasets at scale to enable better data reuse that can facilitate new discoveries. The task of curating and annotating GEO datasets involves the identifying and mapping of biological entities such as genes/proteins, drugs/small-molecules, diseases, and cells/tissue-types at both the dataset and sample levels. Such mapping needs to be done to relevant community-accepted controlled vocabularies such as specialized ontologies available from the National Center for Biomedical Ontology (NCBO) BioPortal (Whetzel et al. 2011) and other community-accepted naming standards. Better annotation of datasets and samples will provide the basis for identifying meaningful biological contrasts among groups of samples, which can then be used for differential expression (DE) analysis. Here, we review recent advances and future perspectives in the process of curating and reprocessing publicly available gene expression datasets from GEO.

## Approaches toward improving curation and annotation of GEO metadata

**Fig. 2:**
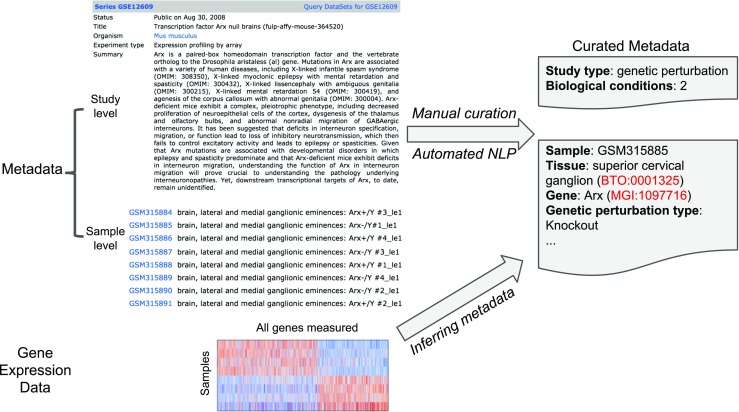
Graphical summary of various curation approaches for further annotating GEO datasets. Metadata and the gene expression data from an example GEO study are shown on the left. Metadata are composed of semi-structured textual annotations supplied by the authors of the dataset at both study-level and sample-level to describe the experimental design of the study, and the characteristics of the samples. The goal of further annotating GEO datasets is to generate structured metadata for each study (top right) and samples (bottom right). Annotations are linked to relevant controlled vocabularies such as ontologies. Three approaches are visualized as arrows: manual curation and automated NLP, both attempt to identify and extract structured metadata from the textual descriptions. In addition, metadata can be inferred from the gene expression data using supervised machine learning approaches

Multiple approaches have been developed for improving the curating of the metadata associated with publicly available studies served on the GEO repository. These methods can be broadly categorized into (1) manual curation, (2) automated natural language processing (NLP), and (3) inferring metadata directly from the gene expression profiles. In the subsequent sections, we describe recent activities within these three categories (Fig. 2).

### Manual curation

Although not perfect, manual curation efforts applied to annotate GEO studies yield high-quality results. However, manual curation does not scale up to cover the tens of thousands of studies that are currently available from GEO. Since GEO, and repositories like it, are expected to drastically grow in the coming years, manual curation is in general not feasible. Crowdsourcing microtasks are projects that consist of a relatively trivial task that requires a large number of participants to complete (Good and Su 2013; Khare et al. 2015). Such an approach is one way to scale up manual metadata curation of GEO datasets. Through a massive open online course (MOOC) on Coursera, we worked together with over 70 participants from over 25 countries to identify and annotate 2460 single-gene perturbation signatures, 839 disease signatures, and 906 drug perturbation signatures from GEO (Wang et al. 2016). The collections of these signatures are served as a web portal called CRowd Extracted Expression of Differential Signatures (CREEDS). CREEDS provides the annotated signatures for query, download, and visualization. A few other similar projects were launched to curate GEO datasets using microtask crowdsourcing strategies. One such project is STARGEO, a website that facilitates the curation of GEO samples with disease phenotypes. The STARGEO project is a manual crowdsourcing curation effort that recruited graduate students to annotate samples with disease phenotypes (Hadley et al. 2017). Another similar effort called OMics Compendia Commons (OMiCC) (Shah et al. 2016) is a community-oriented framework that enables biomedical researchers to collaboratively annotate gene expression datasets and samples. OMiCC is also equipped with a web interface that lets users perform meta-analyses including differential expression analysis.

The manually curated GEO datasets facilitated the reanalysis of multiple related datasets to reveal novel biological insights. For instance, by clustering the curated signatures from genetic perturbation and diseases, we found multiple myelodysplastic syndrome (MDS) signatures from CD34+ cells that cluster with *ERBB2* overexpression signatures from MCF10A cells. Such co-clustering suggests that the upregulation of *ERBB2* and related pathways may play a role in MDS (Wang et al. 2016). Another example is the meta-analysis of inflammatory bowel disease (IBD) signatures across multiple independent studies, curated by the OMiCC platform. This analysis discovered that several peroxisome proliferator-activated receptors (PPARs) are lowly expressed in Crohn’s disease (Shah et al. 2016).

While manual curation through crowdsourcing produces, in general, high-quality annotations, this approach has other drawbacks besides lack of scalability. Curators make mistakes and produce inconsistent annotations in borderline cases (Good and Su 2013; Khare et al. 2015). While this can be resolved through a double-blinded review process, having multiple curators annotate the same datasets increases the burden on the curation task many folds. For the CREEDS project, we had to spot check all entries and remove contributors that produced annotations with high error rates. Another approach to deal with errors made by manual curators is benchmarking. For instance, to validate the quality of the extracted signatures from STARGEO (Hadley et al. 2017), the authors showed that the DEGs from the meta-analysis of curated breast cancer datasets are comparable to signatures automatically generated from The Cancer Genome Atlas (TCGA) resource (The Cancer Genome Atlas Research N et al. 2013). Overall, manual curation efforts produce valuable resources to enable the systems pharmacology community.

### Automated natural language processing

Applying natural language processing (NLP) techniques such as named-entity recognition (NER) and document classification to the textual descriptions of GEO studies is an attractive alternative for curating GEO metadata manually. NLP has been intensively applied to extract structured elements from the free-text of biomedical research publications over the past two decades (Huang and Lu 2016). Within this domain, NER is central. The goal of NER is to identify biological entities of interest, including genes, chemical/small-molecule/drug, disease, cell type, and tissue terms from free-text. Once key terms are identified, document classification models can be trained, using, for example, manually curated samples, to identify perturbation and control samples from GEO using labeled features from text identified by NER. Similarly, such document classification models can be trained to predict the themes of the datasets, including the specific drug treatment, disease model, or the genetic perturbation from the provided descriptions. We used the collection of the manually annotated CREEDS signatures metadata as a training set to train a document classifier for extracting the themes of the datasets from the entire GEO repository (Wang et al. 2016). Subsequent studies further improved NLP-based pipelines by enabling manual adjustments to the automatically curated gene expression datasets. For instance, GEOracle implements a machine learning (ML) classifier that identifies perturbation and control samples from GEO using textual features. It automatically tags samples as perturbation and controls to construct signatures. Importantly, it provides users with the ability to manually adjust the automated selection through a web interface (Djordjevic et al. 2017). Other related work attempted to improve the general quality of the metadata associated with each sample and each GEO study. The leading effort is MetaSRA (Bernstein et al. 2017), a resource that normalized and improved the metadata from SRA. To achieve this, manual annotation of metadata applied to a small subset of SRA was carried out using ontologies for creating a training set. Then, by applying a computational model that implements a data structure called a Text Reasoning Graph, metadata labeling was automatically assigned to the remaining samples.

### Inferring metadata from gene expression profiles

In addition to enriching and normalizing textual descriptions manually or automatically by examining the existing metadata, one can also leverage the information from the gene expression data itself to infer the metadata for curation. Given high-quality annotated gene expression profiles as a training set, ML models can be implemented to automatically identify the metadata from the gene expression profiles. For instance, various algorithms, including URSA (Lee et al. 2013), CIBERSORT (Newman et al. 2015), and xCell (Aran et al. 2017), were developed to predict cell types using gene expression data. Predicted cell types from such algorithms can be integrated with NER methods to corroborate the cell type terms recognized by NER to improve the accuracy of cell-type prediction algorithms directly from data. In the same way, other metadata elements can be predicted directly from the expression data. For example, the automated label extraction (ALE) (Giles et al. 2017) platform was used to impute the age, gender, and tissue type of samples from GEO using the expression data alone. Similarly to ALE, phenotype prediction of processed RNA-seq samples (Ellis et al. 2018) was implemented with ML methods trained using annotated samples from TCGA (The Cancer Genome Atlas Research N et al. 2013) and GTEx (Lonsdale et al. 2013). Another effort that utilized the Center for Expanded Data Annotation and Retrieval (CEDAR) framework (Panahiazar et al. 2017) tested the ability of a classifier to predict few basic common structured metadata elements such as cell type, organism, and platform from GEO samples.

## Future perspectives

### Further improving the curation of GEO datasets with deep and active learning

Current efforts in curating and annotating GEO datasets have exploited the information from both the textual descriptions and the gene expression profiles with manual crowdsourcing and automatic ML/NLP approaches. However, there is still room for further improving both the accuracy and the throughput of such curation tasks. Recent breakthroughs in NER were introduced by the application of deep learning (DL) for this task (Lample et al. 2016; Chiu and Nichols 2015). Due to the significant improved performance, such methods are currently considered the state-of-the-art. Deep neural network implementations of NER typically start with a word embedding layer that maps word tokens to low dimensional vectors that represent the meaning of the words learned from a large corpus using algorithms such as word2vec (Mikolov et al. 2013) and GloVe (Pennington et al. 2014). These word vectors are next connected to various long short-term memory (LSTM) or convolutional neural network (CNN) layers. Then, predictions can be made for each word token, suggesting whether the token is a start, a middle, or an end of a valid named-entity, or is an irrelevant token. The aforementioned state-of-the-art DL-based NER approaches have not been widely applied to biomedical data curation projects yet, perhaps with one exception (Habibi et al. 2017). In a recent study (Habibi et al. 2017), it was demonstrated that a deep neural network (DNN) model, specifically LSTM-Conditional Random Field (CRF) (Lample et al. 2016), outperforms domain-specific models with hand-crafted features in five biomedical NER tasks on 33 datasets. It would be promising to adopt the state-of-the-art deep NER algorithms, and train them on large biomedical corpora such as full-text articles from PubMed Central (PMC) to improve the accuracy of the mapped biological entities.

Another future direction to boost the quality and efficiency of the data curation task of GEO datasets is to develop a hybrid approach of manual and automated curation with active learning (AL). AL is a meta-algorithm for ML that learns to intelligently select examples (data points) for the underlying supervised ML algorithm to train and generalize more efficiently (Cohn et al. 1994). AL is particularly suitable for situations when unlabeled data is abundant and manual labeling is too expensive and time-consuming. AL algorithms attempt to overcome the lack of labeled data by asking human curators to aid with the labeling. The method strategically selects a subset of the data that needs labeling to maximally improve the model performance with minimal labeling requirement. This allows the ML algorithm to improve dynamically while reducing the effort necessary of the human curator (Krishnakumar 2007; Settles 2010). AL methods have been shown to achieve improved performance in similar crowdsourcing settings (Mozafari et al. 2014).

### GEO dataset submission system with improved metadata standardization and validation

To prospectively improve the annotation quality of future datasets that will be deposited into GEO in the coming years, it would be a benefit to create a data and metadata submission system implemented with metadata standardization and validation capabilities. It is feasible to implement web-based submission forms with metadata fields using various minimum information standards (Taylor et al. 2008) such as Minimum Information About a Microarray Experiment (MIAME) (Brazma et al. 2001). These fields can validate user input using external ontologies to ensure the accuracy of the deposited metadata. For instance, small molecule compounds used in a specific study can be validated by their chemical structure representation through UniChem (Chambers et al. 2013). Such mappings would enable cross-referencing to major public chemical databases to enrich the annotations by providing additional annotations, such as mechanism of actions, targets, disease associations, clinical phase status, and synonyms. It has been shown that such data submission systems, with deep metadata annotations that utilize established terminologies and ontologies, contribute to interoperability and reusability of the data (Stathias et al. 2018).

### Toward making GEO datasets more FAIR

Recently, the findable, accessible, interoperable and reproducible (FAIR) guiding principles have been proposed to improve the groundwork needed to support the reuse of scientific data (Wilkinson et al. 2016). The ultimate goal of curating publicly available gene expression datasets is to make repositories such as GEO more FAIR. With the improved metadata annotations, GEO datasets will be more findable by both humans and machines through FAIR-compliant search engines such as the recently developed DataMed (Ohno-Machado et al. 2017; Chen et al. 2018) and Google DataSet Search (https://toolbox.google.com/datasetsearch). These search engines are powered by machine readable metadata that is hosted on dataset landing pages by the data repository using standards such as schema.org (Guha et al. 2016). Advances in web technologies also enable better interoperability between application programming interfaces (APIs). For instance, the BioThings APIs (Xin et al. 2018) can be cross-linked via JavaScript Object Notation for Linked Data (JSON-LD), a data format encoding semantically precise Linked Data, to enable automated knowledge extraction pipelines without having to specify the individual API endpoints and the returned data structures. The use of such technologies for building web services enables better interoperability, and can benefit the integration of GEO datasets with other resources and tools. For example, a researcher will be able to perform a drug-repurposing pipeline by simply specifying a disease of interest, to receive a ranked list of drugs as potential therapeutics through these web-services APIs. This pipeline will start by finding disease-related gene expression signatures, and then identify consensus DEGs through the API serving the annotated GEO datasets, which can then be applied as input for another API that serves drug repurposing queries such as those provided by the applications L1000CDS^2^ (Duan et al. 2016), L1000FWD (Wang et al. 2018a), or clue.io (Subramanian et al. 2017) to retrieve a ranked list of drugs and compounds predicted to reverse the disease signature.

While the curation of metadata and the unified metadata models are important, optimal and uniform data processing pipelines, such as Recount2 (Collado-Torres et al. 2017), ARCHS4 (Lachmann et al. 2018), RNAseqDB (Wang et al. 2018b), and Toil Recompute (Vivian et al. 2017) are also vital for the reusability of the processed gene expression datasets. It is necessary to develop benchmarking strategies for processed datasets from different experimental and computational pipelines. For example, by comparing the consistency between transcription factor knockout and knockdown experiments with ChIP-seq studies that profiled the same transcription factors, we can evaluate the quality of RNA-seq alignment algorithms (Lachmann et al. 2018), calibrate the calling of genes from peaks for ChIP-seq studies, or benchmark methods for differential expression analysis (Clark et al. 2014).

Public gene expression data repositories such as GEO harbor enormous capacity for knowledge discovery. Outstanding progress has been achieved in developing methodologies and tools to facilitate the improved curation and reuse of those datasets in the past few years. However, there is still opportunity to develop better approaches to further advance the quality of GEO’s metadata and data. With the FAIR guiding principles, the resultant improved curated public gene expression datasets will be integrated into an ecosystem of biomedical datasets and knowledge-bases for advancing biological discovery and for accelerating therapeutics development.

## Abbreviations

NCBI: National Center for Biotechnology Information
GEO: Gene Expression Omnibus
SRA: Sequence Read Archive
DE: Differential expression
DEG: Differentially expressed genes
NCBO: National Center for Biomedical Ontology
MOOC: Massive open online course
ML: Machine learning
NLP: Natural language processing
NER: Named-entity recognition
CEDAR: Center for Expanded Data Annotation and Retrieval
LSTM: Long short-term memory
CNN: Convolutional neural network
CRF: Conditional random fields
AL: Active learning
PMC: PubMed Central
FAIR: Findable, accessible, interoperable and reproducible
API: Application programming interface
JSON-LD: JavaScript Object Notation for Linked Data
